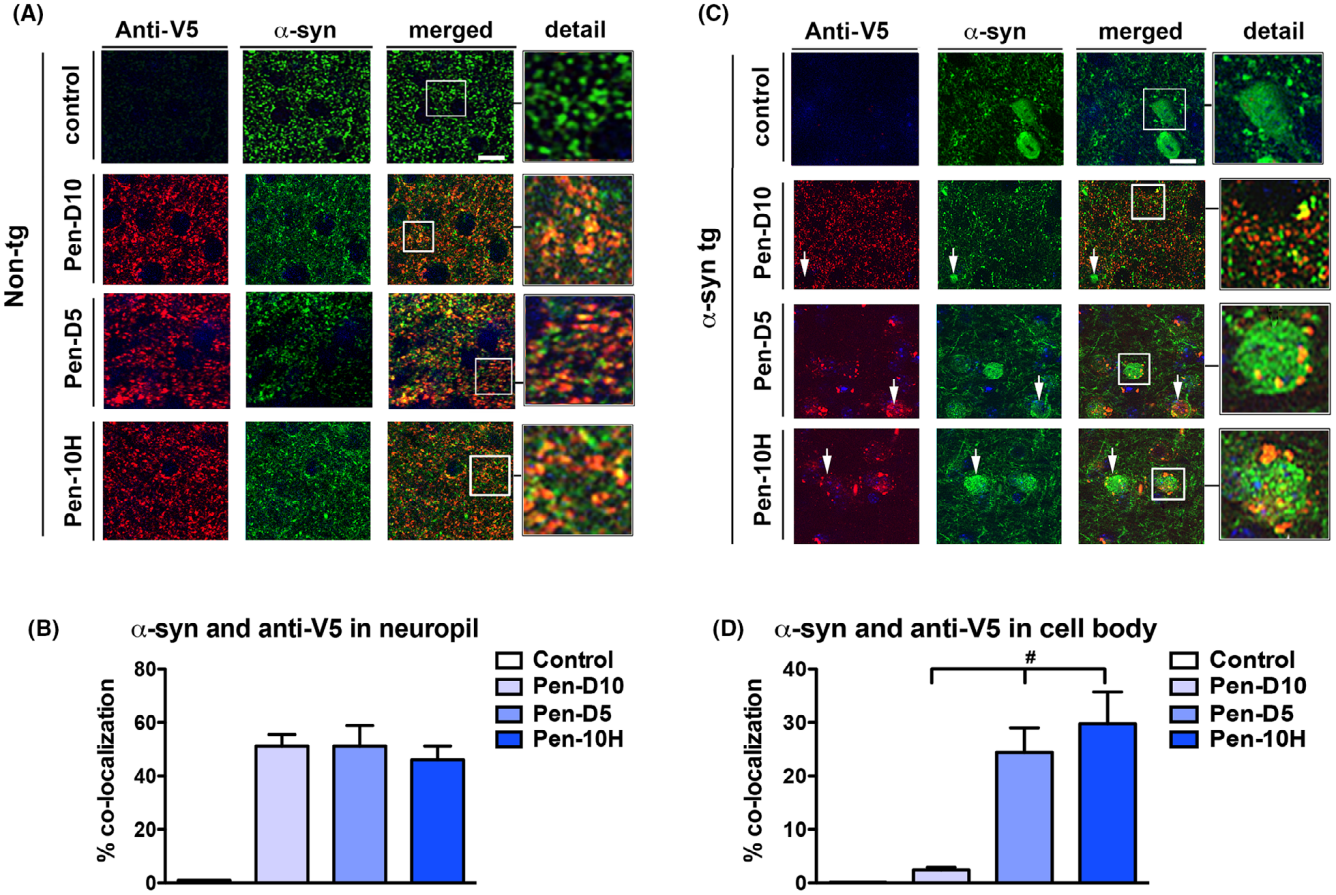# Correction to α‐synuclein conformational antibodies fused to penetratin are effective in models of Lewy body disease

**DOI:** 10.1002/acn3.52266

**Published:** 2025-01-27

Annals of Clinical and Translational Neurology 2016; 3(8): 588–606. doi: 10.1002/acn3.321

The authors regret that an error occurred during the assembly of Fig. 1A and C where the incorrect panel was used to represent the non‐tg Pen‐D5 and the α‐syn tg control condition. The corrected figure is provided in the attachment. We apologize for this error.

**Figure 1:**